# Challenges in management of refractory pain and sedation in infants

**DOI:** 10.3389/fphar.2023.1259064

**Published:** 2024-01-03

**Authors:** Alexandra Oschman, Karishma Rao

**Affiliations:** ^1^ Department of Pharmacy, Children’s Mercy Hospital, Kansas City, MO, United States; ^2^ Division of Neonatology, Children’s Mercy Hospital, Kansas City, MO, United States; ^3^ University of Missouri Kansas City School of Medicine, Kansas City, MO, United States

**Keywords:** delirium, infant analgesia, NICU, infant sedation, opioid-induced hyperalgesia

## Abstract

The survival of preterm infants continues to improve, along with an increased in neonatal intensive care unit (NICU) management of chronic infants who are medically complex infants who have prolonged hospital stays, sometimes up until 2 years of age. Despite advances in neonatal and infant care, the management of pain and sedation in chronic NICU patients continues to be a challenge. Challenges such as development of appropriate pain, sedation, and withdrawal scales along with unfamiliarity of the NICU care team with pediatric disease states and pharmacotherapy complicate management of these patients. Opioid induced hyperalgesia (OIH) and delirium may play a large role in these refractory cases, yet are often not considered in the NICU population. Drug therapy interventions such as gabapentin, ketamine, risperidone, and others have limited data for safety and efficacy in this population. This article summarizes the available literature regarding the evidence for diagnosis and management of infants with refractory pain and sedation along with the challenges that clinicians face when managing these patients.

## Introduction

Neonatal pain had been underrecognized for decades. The pervasive belief was that neonates and preterm infants could not experience pain as intensely as adults due to an immature central nervous system. Furthermore, as they could not verbalize pain nor recollect painful memories, their physiological responses to nociception were largely misunderstood and undertreated. This contributed to the unfortunate reality that until the late 1980s this population often underwent surgery with limited or no pain management (Anand and Hickey, 1987; Hall and Anand, 2014). It was Anand, Phil and Hickey’s landmark article that challenged these archaic beliefs, leading to the significant evolution of pain and sedation management in neonates that we have witnessed in the past few decades (Anand and Hickey, 1987). A multimodal approach consisting of nonpharmacological and pharmacological management of pain and sedation is now considered ideal when indicated (Donato et al., 2019). Nonpharmacological methods include but are not limited to swaddling, sucking, and skin to skin care (McNair et al., 2019). Pharmacological management options are varied as well consisting of non-opioid analgesics, opioid analgesics for pain management and benzodiazepines, α2 agonists* for sedation management in neonates (Donato et al., 2019).

Despite these advances, pain and sedation management in this population continues to remain a challenge. NICUs across the country now house a diverse patient population consisting of neonates less than 700 g combined with a large cardiac population as well as chronic, long-term patients, sometimes even up to 2 years of age. This changing demographic of NICU populations compared to the traditional NICU population complicates appropriate assessment and treatment of pain and sedation due to the wide variety in patient age and diagnosis. Additionally, the pain and sedation scales used in Neonatal Intensive Care Units (NICUs) to determine optimal levels of drug therapy were originally developed to guide management in a different demographic of neonates and infants than what some centers see now. In addition, there is limited data validating their use in neonates and infants; particularly infants 6–24 months of age. The impact of different gestational ages, genetics, critical illness and organ dysfunction on drug metabolism and exposure is also poorly understood. As such there is a lack of robust dosing data and long-term outcomes data to guide management. While the use of analgesics and sedatives in any patient population is never without risks, these risks are amplified in neonates and infants by these unique challenges.

In this article, we hope to highlight the existing barriers to adequate pain and agitation assessment in the neonatal and infant population, focusing on describing the oft lesser understood entities of the neonatal pain and sedation spectrum such as hyperalgesia, refractory pain and delirium. We conducted a thorough review of the literature with a special focus on opioid-induced hyperalgesia and delirium in infants. Animal studies were included when human studies were not available or needed to support a hypothesis. Databases referenced included PubMed and Medline. These databases were searched from inception through 30 May 2023.

## Pain, sedation, and withdrawal scales

An optimal and reliable pain and sedation scale is paramount to ensure appropriate management. Currently, there are more than 40 pain assessment tools that have been developed for neonates, yet only 5 of these scales have been determined to be the most appropriate for neonates and infants (Smith-Parrish et al., 2022). Of these, only 2 can assess both pain and sedation: the Neonatal Pain, Agitation, and Sedation Scale (N-PASS) and the COMFORT scale.

In order to accurately assess pain and sedation, tools need to be available that are valid and reliable for the clinical situation as well as the patient population being assessed. There are a variety of clinical situations that warrant management like procedural pain, post-operative pain, continuous pain due to chronic conditions, pain due to ventilator support, acute pain, etc., but there is little data available on whether the various pain and sedation scales utilized in neonates and infants were correctly implemented for the specific type of pain, sedation, or patient population (Olsson et al., 2021). Olsson et al. (2021) conducted a literature review that included 352 trials with 22 different pain scales, to evaluate the characteristics and reporting of pain scales in infants. Very few of the studies reported on ongoing pain (4.3%); procedural pain was the most common type being studied (91.8%). The most common pain scale assessed was the Premature Infant Pain Profile (44%), followed by the Neonatal Infant Pain Scale (NIPS - 24%). The N-PASS was used in 3% of the 352 studies. NIPS was the second most used scale for ongoing and post-operative pain, yet it is only validated for procedural pain (Olsson et al., 2021).

The American Academy of Pediatrics currently recommends the N-PASS which is also the most commonly utilized scale to assess pain and sedation in the NICU population (COMMITTEE ON FETUS AND NEWBORN and SECTION ON ANESTHESIOLOGY AND PAIN MEDICINE, 2016). However, the utility of N-PASS across all NICU patients today may be challenging as it was originally only validated in a small set of NICU patients very different from the population seen today (Morgan et al., 2020). Hummel et al. (2008) published on the reliability and validity of the N-PASS on acute and chronic pain. The study for prolonged pain included 72 observations in 46 ventilated infants, up to 100 days of age and concluded that the study was a good starting point to show that the N-PASS is a useable, reliable, valid tool (Hummel et al., 2008). But the presence of disease states such as bronchopulmonary dysplasia, pulmonary hypertension, or if patients had multiple diagnoses was not mentioned.

While the N-PASS scale assesses for sedation, most published literature assessed the utility of N-PASS for pain management, resulting in limited data on its effectiveness for sedation management in the now wide range of NICU sub-populations. Most of the literature for N-PASS does include a wide range of gestational ages in mechanically ventilated infants. But there is a paucity of data for assessing adequate sedation or pain in non-mechanically ventilated patients. Morgan published a comprehensive analysis of available N-PASS literature to attempt to assess the validity and reliability of the N-PASS scale (Morgan et al., 2020). Of the 29 articles included in their analysis, 3 measured sedation, and 7 measured both pain and sedation. Sedation in these articles was assessed in preterm, late preterm, and term neonates and infants, primarily in mechanically ventilated patients. Only one of the studies included patients up to 36 months of age (Morgan et al., 2020). Hummel analyzed 40 subjects, up to 36 months of age in the post-operative recovery period. Scores for sedation were conducted with the N-PASS, the University of Michigan Sedation Scale (UMSS), and the Face, Legs, Arms, Cry, and Consolability (FLACC) scale (Hummel et al., 2008). Correlation between the N-PASS sedation scale and UMSS and FLACC were assessed by Cronhach’s alphas and found acceptable consistency with alphas from 0.853 to 0.938. They did find that scores were more reliable if vital signs were not evaluated as part of the sedation score. Specifically, heart rate had a significant effect on the reliability of the N-PASS score (Hummel, 2014). Limitations of these results are largely that while they did evaluate older NICU infants, it was in the immediate post-operative period in the post-anesthesia care unit and this may not be reflective of chronic sedation needs in an older infant in the NICU. Morgan and others also concluded from their literature review that while the N-PASS appears to be the most reliable and valid scale available for pain and sedation assessment, consideration for the many different sub-populations of NICU patients is paramount (Morgan et al., 2020).

The above-mentioned reviews highlight that despite the availability of multiple pain and sedation scales there are limitations to their applicability which limits the clinician’s ability to appropriately assess pain and sedation in the NICU population in different clinical situations. Overassessment may result in unnecessary use of pain and sedative medications, the majority of which come with potential short- and long-term adverse effects (Maxwell et al., 2019). Conversely, underassessment of pain and sedation can lead to long lasting consequences such as altered pain sensitivity and behavioral abnormalities (COMMITTEE ON FETUS AND NEWBORN and SECTION ON ANESTHESIOLOGY AND PAIN MEDICINE, 2016; Maxwell et al., 2019).

## Refractory pain and sedation

Chronic infants have multiple confounding factors that can contribute to pain and agitation that can be difficult to delineate. Many of the symptoms of pain (tachycardia, excessive crying, facial grimacing, vigorously bending arms and legs, along with increased respiratory rate) can also be symptoms of agitation, or their underlying disease state, particularly with bronchopulmonary dysplasia or cardiac patients. The difficulty in delineating pain versus agitation symptoms additionally makes application of pain and sedation scoring tools challenging as the tools have a lot of overlap in assessing for the same symptoms. This makes it difficult for the bedside observer to delineate the symptom as a pain or agitation symptom. The optimal approach to managing pain and sedation in infants in unknown (McPherson et al., 2021). There are multiple reasons patients may require pain and sedation management (post-operative, mechanical ventilation, intubation, etc.), some of those being more straightforward to delineate symptoms such as post-operative pain. There are published recommendations on the prevention and management of pain for these conditions (minor procedures, mechanical ventilation, post-operative, and major procedures) (COMMITTEE ON FETUS AND NEWBORN and SECTION ON ANESTHESIOLOGY AND PAIN MEDICINE, 2016). Focusing on refractory pain and sedation, it is important to look for underlying causes as well as optimizing drug therapy, despite no data to make strong recommendations.

Chronic pain is not a concept that is frequently considered in the NICU population, but may be imperative to evaluate in the older, chronic infant. In pediatric patients, chronic pain is defined as recurrent or persistent pain that extends longer than the anticipated healing time, generally considered more than or equal to 3 months of pain (Friedrichsdorf and Goubert, 2020). Types of chronic pain in pediatrics can be classified as nociceptive pain, neuropathic pain, and idiopathic pain (Watson, 2020). These pain types are not commonly considered in the infant population and may be reasons for what appears to be increasing pain or agitation in patients who previously had been stable on their pain and sedation regimens. Commonly utilized medications in the NICU population for pain and sedation include morphine, fentanyl, benzodiazepines, and dexmedetomidine (Donato et al., 2019). The treatment choices commonly utilized in the NICU for patients with chronic pain may further complicate management by contributing to the development of opioid-induced hyperalgesia, antinociceptive tolerance, and dependence. Limited treatment options for management of pain and sedation in the NICU population can additionally make management of patients challenging, particularly those patients who appear to not respond despite relatively high doses of morphine, midazolam, and dexmedetomidine. Thoughtful decision making in the use of analgosedatives are imperative.

Amigoni and others suggest optimizing analgesia utilizing opiates for analgesia and utilization of alpha agonists for sedation (Amigoni et al., 2022). Due to the effect of benzodiazepines and their negative effect on neurodevelopment in patients less than 3 years of age along with the direct, dose-dependent association with the development of delirium, benzodiazepines are recommended as second line for sedation. Third line drug therapy options include medications that act upon GABA or N-methyl-D-aspartate (NMDA) receptors. The implementation of a standard analgesia and sedation protocol has been found to decrease exposure to these medications which can show a reduction in the development of tolerance and refractory pain and sedation (Neunhoeffer et al., 2017).

Defining refractory pain and sedation is difficult. Various definitions include the use of more than three sedative drugs, presence of inadequate sedation that lasts more than 2 h, the need to increase doses to higher than the 90th percentile from the usually starting dose, or the need to administer neuromuscular-blocking agents to control ventilation comfort (Amigoni et al., 2022). This definition when applied to the NICU population would indicate that refractory pain and sedation in the NICU can be frequent, particularly in chronic ventilated patients. The consequences of ineffectively managing refractory analgosedation are not benign and include delirium, unplanned extubation, unplanned removal of invasive devices, and paradoxical responses to sedation.

Management of refractory pain and sedation is challenging due to the limited literature regarding management and the safety of those agents in the neonatal and infant population. A component of refractory pain may be attributable to the development of hyperalgesia. The development of hyperalgesia is a result of exposure to opioids where a state of nociceptive sensitization has occurred. The concept of opioid-induced hyperalgesia (OIH) has regained popularity and describes a condition where there is worsening pain sensitivity without a new injury or exacerbation of an old injury when chronically exposed to opioids (Doyle et al., 2020). Patients with OIH may present with increasing pain or sedation needs, despite previously adequate doses, without a reason for increasing analgesic needs. OIH was first described in the literature in 1942 but has limited literature regarding a clear definition for diagnosis, when or who it may develop in, whether it’s related to acute or chronic opioid exposure, or if the dose of opioid plays a role.

The exact mechanism of OIH not well understood. There are several potential mechanisms for OIH, which include stimulation of the excitatory amino acid neurotransmitter system, sensitization of pronociceptive pathways involving the glutaminergic system, and involvement in other signaling systems (Doyle et al., 2020). The effect of activating these pathways results in hyperalgesia, myoclonus, and other negative opioid effects (Peters et al., 2006). Opioid receptors may not be involved in OIH as mice with knockout genes for a variety of opioid receptors still developed OIH. These mice did still experience hyperalgesia, supporting the findings of Peters, et al. that morphine 3-glucuronide binds to other receptors and contributes to morphine-induced hyperalgesia (Peters et al., 2006; Swartjes et al., 2012). When morphine is converted to its two metabolites (morphine 3-glucuronide and morphine 6-glucuronide), greater than 50% of morphine is converted to morphine 3-glucuronide, with approximately 10% converted to morphine 6-glucuronide. Morphine 3-glucuronide is associated with neuroexcitability and actions opposing the analgesic effect of morphine 6-glucuronide. Accumulation of both metabolites can be seen in patients with renal insufficiency, which can maybe see in premature neonates and infants as their renal function is immature (Dean, 2004). Due to the negative effects of morphine 3-glucuronide, the use of morphine, which is traditionally a preferred medication for pain and sedation in the NICU population, may contribute to OIH and the appearance of refractory pain. N-methyl-D-aspartate (NMDA) receptors are a glutamate receptor that plays an important role in anesthesia and analgesia. NMDA receptors have multiple roles in the body, including effects on brain development, excitotoxicity, peripheral nerve pain, and pain sensitization (Petrenko et al., 2003). Continuous activation of NMDA receptors has been shown to result in increased sensitivity to pain (Petrenko et al., 2003; Angst and Clark, 2006; Doyle et al., 2020). Animal studies assessing OIH found that utilization of NMDA receptor antagonists were effective in blocking or partially blocking the development of OIH (Angst and Clark, 2006; Tompkins and Campbell, 2011).

### Management strategies

There are limited management strategies for OIH, primarily due to the lack of literature and the ability to diagnose OIH. Studies assessing opioid switching has not been shown to be effective for OIH or for decreasing opioid exposure (Tompkins and Campbell, 2011; Amigoni et al., 2022). Decreases in opioid doses may sometimes be helpful, but literature is lacking. The majority of data suggests that the addition of NMDA antagonists show the most promise (Tompkins and Campbell, 2011). Many questions still exist regarding OIH, including how to diagnose it, how to manage it, and is there a way to decrease risk of development of OIH.

Whether treating OIH or refractory pain and sedation, ketamine, gabapentin, and methadone have been suggested in a variety of sources as potential options (Tompkins and Campbell, 2011; Murphy et al., 2021; Amigoni et al., 2022). Ketamine is a unique, rapid-acting sedative with analgesic properties. It works by non-competitive antagonism of the NMDA receptor. Ketamine does have some unique properties compared to other analgosedative options that can make it a good choice in hemodynamically unstable patients, or those where the bronchodilation effect of ketamine may be beneficial. The BPD patient population may represent a population where these effects would be beneficial. The majority of available ketamine literature for OIH is from the post-operative pediatric or adult population. In a randomized, double-blind, placebo-controlled trial in 130 adult spinal fusion patients, the addition of 0.03 mg/kg/hr of ketamine intraoperatively followed by 0.1 mg/kg/hr for 48 h post-operatively lowered intravenous hydromorphone requirements by a median of 2 vs. 4.6 doses/day in the ketamine group. Both groups received methadone 0.2 mg/kg intraoperatively. In addition, the use of oral opioids post-operatively was lower in the ketamine group on day 1 and day 3 (0–3 vs. 0–8 tablets; *p* = 0.002) (Murphy et al., 2021). Kharasch and Clark (2021) commented on the results of the Murphy results, stating that the magnitude of the effect of ketamine seen was unusually large, and may represent a boosted effect of methadone. A potential explanation for the boosted effect seen may be that ketamine helped to ameliorate OIH. The majority of patients in the Murphy study were on chronic opioids prior to surgery and ketamine may have worked via indirectly mitigating opioid-induced sensitization. Kharasch also suggest that as methadone has NMDA antagonist activity that the combination of methadone and ketamine could be an effective combination to manage post-op pain control and reduce OIH via NMDA antagonism. Despite the lack of literature supporting the use of ketamine in pediatric patients, it has been recommended for use for pediatric ICU patients with refractory analgosedation (Amigoni et al., 2022).

Gabapentin is a structural analog of Gamma-aminobutyric acid (GABA). It’s mechanism of action is not fully elucidated, though one mechanism of action is the decreased release of GABA, resulting in less neuronal hyperactivity. It’s varied mechanisms allow for its use in a variety of conditions such as dystonia, irritability, neuropathic pain, post-operative pain, epilepsy, and visceral hyperalgesia. Use of gabapentin as an adjunct for pain allows for weaning or discontinuation of other neurosedatives. The use of gabapentin in NICU patients was associated with an 80% decrease in the number of patients receiving 3 or more neurosedatives (Burnsed et al., 2020). These results have been replicated in other centers in medically complex infants (Sacha et al., 2017).

Methadone is an opioid consisting of a racemic mixture, where the D-isomer functions as an NMDA receptor antagonist. Methadone’s place in therapy has generally been for long term weaning of opioids in chronic infants. The effect of methadone on NMDA receptors and it’s potential to decrease hyperalgesia has led to an increase it its use. Methadone does have a long half-life that can be appealing to provide analgesia in chronic infants with long term pain and weaning requirements who are stable on the opioid doses. This may be preferential to long term continuous infusion medications which require line access, increasing the risk of infection and line complications.

## Safety of analgosedatives in infants

The majority of NICU patients are exposed to at least one opioid (fentanyl or morphine) or benzodiazepine (midazolam, clonazepam, or lorazepam) during their hospital stay (Puia-Dumitrescu et al., 2021). In a retrospective follow up of 936 extremely preterm infants, infants exposed to both opioids and benzodiazepines for greater than 7 days had lower Bayley Scales of Infant Development-Third Edition (BSID-III) scores compared to infants without exposure (median [interquartile range] motor score, 85[73–97] vs. 97[91–107]) at 2 years follow-up (Puia-Dumitrescu et al., 2021). While morphine is generally considered more benign that the use of benzodiazepines, use of opioids are not without consequence. The NOPAIN and NEOPAIN studies showed that morphine use can increase duration of mechanical ventilation, delay tolerance of enteral feedings, and affect muscle tone development. Long term follow up studies are mixed, likely representative of the broad range of concomitant disease states that additionally contribute to increased morbidity (Anand et al., 1999; Anand et al., 2004).

Benzodiazepines have negative effects in the developing neonatal brain. Proposed mechanisms of injury include hemodynamic adverse effects from midazolam. Midazolam may cause hypotension in up to 45% of preterm infants. This hypotension can result in decreases in oxygen saturation and cerebral oxygenation and blood flow velocity. Benzodiazepines have also been shown in models to cause widespread neuroapoptosis and decreased neurogenesis with exposure. The actions of benzodiazepines on the GABA receptors is the likely mechanism of neuroapoptosis (McPherson et al., 2021). GABA and NMDA systems play an important role in neurodevelopment, in developing the connection and communication between neurons. In the developing brain, neuroapoptosis occurs when there is an absence of binding on GABA and NMDA receptors (Andropoulos, 2018). Binding to GABA and NMDA receptors blocks normal neurotransmission. The synaptic deprivation that occurs leads to activation of an intrinsic neuroapoptotic cascade, resulting in mitochondrial disruption. This disruption leads to an increase in caspase-3, which results in apoptosis (Andropoulos, 2018).

Two randomized controlled studies have evaluated the effect of benzodiazepine use in the neonatal population. The NOPAIN trial included 67 preterm infants, who were randomized to receive midazolam, morphine, or placebo. There was a difference in the incidence of severe intraventricular hemorrhage, periventricular leukomalacia or death in the midazolam group compared to morphine (32% vs. 4%, *p* = 0.03). Though data regarding long-term neurodevelopmental effects of benzodiazepine therapy does not exist, the use of benzodiazepines in the neonatal time frame has declined due to the results of the NOPAIN study (Anand et al., 1999).

There is a lack of literature on the long-term effects of ketamine or gabapentin when utilized in neonates and infants. Literature is clear on the negative effects of benzodiazepines on the developing brain. The neuroapoptosis seen with benzodiazepines are likely due to the effect on GABA receptors (McPherson et al., 2021). Gabapentin works via GABA mediation, so it would be feasible that gabapentin may have similar deleterious effects on neurodevelopment. While data with gabapentin is lacking for short and long-term effects, there is significant data regarding the effects of ketamine, though all in animal studies. Exposure of infant rats to anesthetic agents is known to trigger apoptosis, that effect is more profound if both GABA and NMDA receptors are involved (Andropoulos, 2018). The highest risk point for use is in the early neonatal period. Long-term learning and memory deficits have been seen with ketamine use and has been replicated hundreds of times in animal models (Yan et al., 2014; Cheung and Yew, 2019). An argument could be made that the chronic NICU patient with refractory pain and sedation is no longer a neonate, however the brain is still developing and could have deleterious effects when exposed to these agents.

Despite the negative effects of benzodiazepines in the neonatal and infant population, benzodiazepines are still utilized to provide sedation in this population. There is a lack of sufficient drugs to provide adequate and safe sedation. Morphine is often utilized in this population to provide pain and sedation. This is not optimal as morphine utilization is also not without negative effects such as dependence and negative impacts on neurodevelopment. Options suggested for use with OIH and refractory analgosedation such as gabapentin, ketamine, and methadone all have limited data regarding long-term effects. However, what data is available would suggest caution should be utilized when choosing to use these agents in the NICU population.

## Delirium

Delirium is the behavioral manifestation of acute cerebral dysfunction. It is defined as a disturbance in attention and cognition per the new *Diagnostic and Statistical Manual of Mental Disorders Fifth Edition (DSM-5)* (Silver et al., 2015). It presents as an acute, fluctuating change in awareness and mental status with disordered sleep wake cycle in the setting of an underlying medical illness (Lipowski, 1987; Silver et al., 2010; Silver et al., 2015). There is extensive literature outlining delirium in the adult population (Ely et al., 2004). There is now a growing body of data on pediatric delirium as well (Groves et al., 2016). However, our understanding and recognition of infant delirium remains severely limited in comparison.

### Pathophysiology

The pathophysiology of delirium is complex and poorly understood. It is a result of interplay between predisposing factors and precipitating factors. Predisposing factors are the background characteristics of the patient, such as, age, cognitive impairment, developmental delay. Precipitating factors are triggers such as hypoxia, hypoglycemia, sepsis, surgery, mechanical ventilation, medications. Any such combination of predisposing and precipitating factors may then ultimately result in altered neurotransmission (Silver et al., 2015; Patel et al., 2017; Wilson et al., 2020). This disruption of normal neurotransmission results in acute changes in mental status, altered consciousness or cognition, and other psychomotor behaviors seen in delirium (Smith et al., 2022).

There are three main subtypes of delirium: hypoactive, hyperactive and mixed. Hypoactive delirium is seen when there is a deficiency of dopamine or an excess of acetylcholine or GABA receptor stimulation. Hyperactive delirium is caused by an excess of dopamine activity and acetylcholine antagonism. Critical illness contributes to the development of delirium through the effect of illness of the neurotransmitter system and the utilization of medications that affects the dopaminergic, cholinergic, and glutamatergic systems (Patel et al., 2017; Smith et al., 2017).

### Risk factors

Risk factors for delirium in pediatric patients include the above mentioned predisposing factors: age ≤2 years of age, developmental delay, pre-existing medical conditions, and severity of illness. Most studies suggest that children with developmental delays are at higher risk for developing delirium during their ICU stay. This is similar to findings in adults where the risk of delirium is higher in those with preexisting cognitive impairment or cerebral atrophy in the setting of a critical illness (Silver et al., 2015; Wilson et al., 2020). This seems to suggest that an atypical brain is predisposed and more vulnerable to the stressors of critical illness. Based on these risk factors, neonates and infants are likely at higher risk for delirium.

Other Pediatric Intensive Care Unit (PICU) specific risk factors include mechanical ventilation, coma, use of benzodiazepines, opioids, anticholinergics, and vasoactive medications. Patients receiving benzodiazepines compared to those that did not have a fivefold higher risk of delirium (Traube et al., 2017). While benzodiazepine use has been highly associated with the risk of delirium, more data regarding specifics of medication use, cumulative dose, route of administration, and delineating that info from other confounders is needed.

### Diagnostic challenges

Delirium is not well recognized as a complication of NICU hospitalization. This is in part due to a lack of awareness or knowledge of the diagnosis itself and partly due to difficulties in diagnosis and assessment.

While the presentation of infant delirium is consistent in definition with the DSM criteria, this can be hard to definitively discern in clinical situations as it is challenging to establish a good baseline in nonverbal and/or developmentally delayed infants. Delirium in infants can present as “refractory agitation” (Groves et al., 2016). Case reports have often described a NICU patient that is critically ill, mechanically ventilated, that becomes “impossible to sedate” and does not respond to escalating doses of sedatives (Groves et al., 2016). Delirium in this age group can also present with subtle developmentally specific signs and symptoms (Silver et al., 2010; Groves et al., 2016) making the clinical diagnosis more challenging.

Lack of robust data and prospective trials in infants also contributes to the uncertainty that many clinicians may experience about the diagnosis of delirium in infants. Given that neonatal pain was difficult to conceptualize for decades by the medical community, it is no surprise that infants experiencing delirium is even harder to imagine. However, we must humbly accept that there is a lot yet that we do not fully understand about the developing neonatal brain and its handling of pain. There is growing evidence to suggest that neonates do experience delirium in the same way as adults and current data supports that pediatric delirium is associated with increased length of hospitalization, increased healthcare costs and mortality (Patel et al., 2017).

### Prevention of delirium in infants

There is limited robust data regarding utilizing on non-pharmacologic interventions such as environmental modifications on the development of delirium in pediatric patients. In adults, decreases in the incidence of delirium was seen with appropriate sleep/wake cycles, family presence and involvement, and comforting objects from home (Smith et al., 2022). Many of the non-pharmacologic interventions are not feasible in the NICU population. A single center study in pediatric patients found that protocolized sedation along with environmental modifications resulted in a decrease in delirium rates by 40% (Simone et al., 2017). Delirium is often triggered by critical illness and other underlying conditions, so identification and management of those etiologies should be undertaken. A common acronym to utilize to remember potential diagnoses is BRAINMAPS (bring oxygen, remove/reduce deliriogenic drugs, patient atmosphere, immobilization, new organ dysfunction, metabolic disturbances, awake, pain, and sedation) (Smith et al., 2022).

### Management of delirium

Increased vigilance and index of suspicion for diagnosing delirium in infants is the first step. Pediatric specific bedside screening tools should be utilized. Specifically, the CAPD tool is an observational screening tool, validated and reliable for detecting delirium in the critically ill infant population of all ages. This tool takes a few minutes to complete and should be completed a few hours into the nursing shift ideally, twice a day (once each shift), to offer a longitudinal assessment of the patient. This tool can be utilized for children 0–21 years of age and is validated in developmentally delayed children was well. A score of 9 or greater is consistent with a diagnosis of delirium (Groves et al., 2016; Patel et al., 2017).

An important step in the management of delirium is to identify the underlying cause and rectify it. This includes antibiotics for sepsis management, addressing hypoxemia, electrolyte imbalances and other modifiable causes. The next step to consider is to eliminate any iatrogenic causes of delirium with special consideration being given to decrease Deliriogenic medications such as benzodiazepines. They are known to stimulate GABAA receptors, contributing to the development of delirium (Turkel et al., 2013a; Patel et al., 2017; Smith et al., 2017). Guidelines in pediatric patients suggest limiting exposure to benzodiazepines along with opioids when possible and implementing a standard sedation protocol (Smith et al., 2022). Several studies have found a decrease or improvement in delirium with reducing benzodiazepine exposure either thought discontinuation or lowered doses (Mody et al., 2018). Consideration can be given to switching to dexmedetomidine which provides sedation through alpha-2 receptor agonism. While dexmedetomidine is suggested as an alternative to benzodiazepine use for sedation, robust studies evaluating its effect on delirium is lacking.

An important aspect of delirium management is the nonpharmacological management and attempts to address patient environment. This includes decreasing noise, minimizing sleep disruptions, clustering cares and creating a stable, familiar environment for the infant. If despite all these efforts, delirium persists, then pharmacological management of delirium may be considered.

Antipsychotics such as haloperidol and atypical antipsychotics are utilized in adult patients with delirium. The use of antipsychotics is not currently recommended in pediatric delirium though the pediatric delirium guidelines do suggest their consideration for refractory delirium (Smith et al., 2022). Quetiapine is an atypical antipsychotic indicated for use in schizophrenia and bipolar disorder, but also has an off-label indication for use of delirium in critically ill pediatric patients. Atypical antipsychotics should be utilized cautiously due to potential neurologic, cardiac, and metabolic adverse effects along with a lack of adult data showing significant improvement in the duration of delirium (Jesus et al., 2020). Dosing of quetiapine for delirium in infants is not well described. Quetiapine is metabolized by cytochrome 3A4, thus likely has decreased metabolism if utilized in young infants.

Groves, et al. published a series of 3 premature infants with delirium. The patients were suspected of delirium due to increasing agitation, inconsolability, altered sleep-wake cycles. Episodes of agitation were not responsive to boluses of morphine or fentanyl. All 3 patients were initiated on quetiapine 0.5 mg/kg/dose enterally every 8 h. After 48–72 h, all 3 patients had improvement in their agitation, allowing for neurosedative weaning (Groves et al., 2016). Other case reports have described the use of risperidone as well. Careful monitoring for QTc prolongation is recommended if initiating atypical antipsychotic medications to treat delirium (Brahmbhatt and Whitgob, 2016). Additional downsides to quetiapine use in pediatric patients is the lack of a liquid dosage form, making accurate dose measurements difficult.

Risperidone is a benzisoxazole atypical antipsychotic that function by affecting multiple receptors including serotonin and dopamine. Risperdone has fewer adverse effects compared to other atypical antipsychotics, but is still associated with prolongation of the QT interval, anticholinergic effects such as constipation, blurred vision, urinary retention, confusion, and agitation. Turkel et al. (2013b) conducted a retrospective review on patients under the age of 4 years old who had delirium and were treated with an antipsychotic medication. A total of 19 patients were evaluated, with an average age of 20.5 months. Olanzapine was utilized in 16 patients and risperidone in 3. Starting doses of both medications were not weight based. Olanzapine doses were either 0.625 mg or 1.25 mg at bedtime or twice a day. Risperidone was dosed at 0.05–0.1 mg at bedtime or twice a day. Improvement in delirium was seen in the majority of patients and significant adverse effects were not seen (Turkel et al., 2013b). More recently, a retrospective review of risperdone use in pediatric delirium patients less than 2 years of age was published on 17 patients. The initial daily dose ranged from 0.1 to 0.25 mg (0.01–0.04 mg/kg) with an average starting dose of 0.02 mg/kg once a day. Approximately 80% of patients required a dose increase during therapy. Max daily doses ranged from 0.1 to 0.5 mg (0.01–0.1 mg/kg) with a median of 0.25 mg/day. Adverse effects were assessed via evaluation of available electrocardiographs to evaluate QTc. Risperidone was found to be safe and effective for management of pediatric delirium (Campbell et al., 2020).

## Discussion

Infants experience many painful procedures routinely in the NICU. The EPIPAIN study outlines many such frequent stressful and painful procedures that neonates may experience daily, like heel sticks or nasal or endotracheal tube suction (Barker and Rutter, 1995; Carbajal et al., 2008). However, pain in infants is poorly understood. The lack of valid, reliable scales for pain and sedation assessment further complicates appropriate management. NICU patient populations have become increasingly heterogenous making it even more challenging to find a scale with wide applicability.

Complicating management of pain and agitation is delineating true pain and agitation from opioid or benzodiazepine withdrawal or other conditions that may manifest with similar symptoms (air hunger, ventilatory asynchrony, hypertonia, feeding intolerance, inflammatory conditions). The lack of well validated narcotic withdrawal assessment scales contributes to both over and under-treatment of withdrawal and affects management of true pain or agitation. Due to this, patients may receive extra pain and sedative medications to treat withdrawal based on subjective and/or physiologic markers, that may not truly be reflective of withdrawal. The modified Finnegan score which was developed for use in neonates exposed *in utero* to opioid substances is utilized widely in NICU patients for iatrogenic opioid and benzodiazepine exposure to assess for withdrawal. Despite the availability of other scales that may be more accurate in the older NICU population such as the Withdrawal Assessment Tool-1 (WAT-1), many centers continue to utilize the modified Finnegan score in an unvalidated patient population.

In addition to the above challenges, new challenges exist when considering chronic pain, sedation, and OIH in this patient population. There is very limited data regarding OIH in neonates and infants, along with a lack of robust diagnostic criteria. More challenging is that the potential drug therapy options have their own challenges as well. Optimal dosing for medications not traditionally utilized in this population is lacking and the varied maturation of metabolic processes would indicate dosing of many of these medications would not be the same as in pediatric patients as the majority of these medications are metabolized by enzymes at 50% of their pediatric/adult capacity at best (Table 1 (Lu and Rosenbaum, 2014)). Not only is dosing not well elucidated, but several of the medication options are also associated or have the potential to cause long term adverse effects. Ketamine has been known to cause neuroapoptosis in neonates and has limited data. Hydromorphone is not commonly utilized in this population and it is unknown what optimal dosing may be. Gabapentin and methadone, while more commonly utilized, still constitutes a small percentage of use and long-term effects are not known.

**TABLE 1:** Maturation of metabolic processes for varied pain and sedative agents (Lu and Rosenbaum, 2014).

	Mechanism of metabolism	Maturation of metabolism
Gabapentin	Not metabolized (100% renally cleared)	-------
Hydromorphone, Morphine	Hepatic glucuronidation; Morphine (UGT2B7)	Adult levels by 2–6 months of age
Ketamine	N-demethylation via CYP3A4 (minor from CYP2B6, CYP2C9), hydroxylation, and conjugation	Varied; CYP3A4 reaches 50% of adult activity at 3–12 months of age
Methadone	N-demethylation via CYP3A4, CYP2D6, CYP2C19, CYP2C9,CYP2D6	Varied rates of maturity; Except CYP2C19; all are at 50% of adult activity or less at 1 year of age
Morphine	Hepatic conjugation; UGT2B7	Adult levels by 2–6 months of age
Midazolam, Risperidone, Quetiapine	CYP3A4	20% of adult activity at a week of age; 50% of adult activity at 3–12 months of age

Delirium poses an additional challenge, as some of the patients that are felt to experience refractory pain and sedation may be experiencing delirium. The pediatric delirium guidelines do provide a good starting point, however, there is a lack of strong recommendations for pharmacologic treatment and many of the non-pharmacologic and preventative options are not optimal in an infant population.

In just a few short decades, we have come a long way in our acknowledgement and management of neonatal pain and sedation. However, we need more studies to help us better understand neonatal pain, to help us better differentiate between an infant’s response to pain versus stressful events (such as diaper changes). These studies can then guide the development of pain and sedation assessment scales in this population with broader applicability even through chronic diseases such as bronchopulmonary dysplasia and/or infants with chronic mechanical ventilation needs. As we begin to understand the impacts of untreated pain on the neonatal brain, we can hopefully do larger clinical trials to cater pharmacological pain and sedation management to specific disease states in neonates as we gather more long-term data on the effects of these medications. Most importantly, we urge neonatal clinicians to recognize that we are currently limited not just by the lack of appropriate tools or data, but we may also be limited by our lack of imagination: we hope that this review offers an opportunity to reflect and consider that an infant’s brain can also experience the entire spectrum of pain and withdrawal as experienced by adults, including OIH and delirium. We hope that readers will recognize the need to monitor, assess and manage OIH, infant delirium and eventually contribute their experience and conduct research in these areas, so we can better care for the developing brain.
